# Most common, burdensome, and worrisome symptoms experienced by people living with early Parkinson’s disease in Germany and the United States—Results from a cross-sectional survey

**DOI:** 10.3389/fneur.2026.1756255

**Published:** 2026-07-08

**Authors:** Simon Messner, Àngels Costa, Todd Carmody, Rebekah Park, Byron Jones, Elisabeth Lucassen, Amelia Hursey

**Affiliations:** 1 Novartis Pharma AG, Basel, Switzerland; 2 Gemic, New York, NY, United States; 3 Parkinson’s Europe, London, United Kingdom

**Keywords:** early-stage, non-motor symptom, Parkinson’s disease, patient-reported symptoms, tremor-dominant

## Abstract

**Background:**

The majority of research on the patient experience of Parkinson’s disease (PD) has focused on the frequency and burden of late-stage symptoms, with less emphasis on understanding symptoms in the earlier phases of the condition.

**Objective:**

This descriptive, cross-sectional study aimed to identify the most common, burdensome, and worrisome symptoms of early-stage PD (ePD).

**Methods:**

An online, self-administered survey was completed by people with ePD (within 5 years of diagnosis) living in the United States and Germany. Descriptive statistics were used to report respondents’ characteristics and responses to the survey questions.

**Results:**

Out of a total of 150 survey participants, 106 were diagnosed with ePD for 0–3 years, while 44 participants were diagnosed for 4–5 years. All respondents reported experiencing tremor, which, together with mobility issues and pain, were most frequently reported as the most burdensome symptoms in both groups. Bradykinesia, mobility issues, memory loss/time lapse, cognitive impairment, and urinary and gastrointestinal problems were reported with a frequency of at least 30% higher in the 4–5-year group.

**Conclusion:**

In the surveyed population, the reported frequency of symptoms such as tremor, mobility issues, and pain was higher among those who were 4–5 years post-diagnosis, and these symptoms were reported as the most burdensome. The frequency and impact of motor and non-motor symptoms should be evaluated in future interventional clinical trials involving people living with ePD.

## Introduction

Parkinson’s disease (PD) is a progressive neurodegenerative disorder affecting approximately 8.5 million people worldwide (1). It is noted as one of the world’s fastest-growing movement disorders. However, its cause remains unknown, and there is currently no cure. PD is characterized clinically by motor symptoms, including tremor, slow movement, and rigidity, and non-motor symptoms such as cognitive impairment, sleep disturbances, and pain (2). Current pharmacological and rehabilitative treatments focus primarily on helping people living with PD manage their daily symptoms, which progress gradually from mild to more severe stages over time. PD predominantly affects elderly people, specifically those aged 60 years and older. It negatively impacts the wellbeing and quality of life of those who receive the diagnosis, significantly affecting their everyday lives and those of their families and potential caregivers (3–6).

Patient experiences in the later stages of PD are well-documented (7–9). Established patient-reported outcomes measures (PROs), such as the Parkinson’s Disease Questionnaire (PDQ-39), or other tools such as the Movement Disorder Society-Unified Parkinson’s Disease Rating Scale (MDS-UPDRS) Parts IB (non-motor) and II (motor) have been widely used in clinical research to gather patients’ subjective experiences and measure changes in their symptoms over time (10). Patient-focused qualitative studies have drawn important attention to significant mobility limitations and access challenges to care, complex caregiving relationships, and difficult end-of-life decision-making faced by many people living with advanced PD (11–14).

However, relatively little is known about patients’ symptom experiences in the early and prodromal stages of PD, and existing PRO tools are limited in their ability to capture symptoms at these stages (10, 15). Early-stage PD (ePD) has generally been defined as Hoehn and Yahr Stage I or II, or as a composite measure of time since diagnosis (i.e., less than 5 years) and/or functional impairment (10, 16). Early PD presents a unique set of needs and challenges (17–21). Motor symptoms are a major cause of reduced quality of life in early and mid-stage PD (22). However, in ePD, non-motor symptoms such as increased occurrences of anxiety, depression, constipation, and sexual dysfunction often precede motor symptoms and significantly impact quality of life (23–28). Due to the sensitive, nuanced, and potentially embarrassing nature of these symptoms, along with individuals’ lack of awareness and knowledge gaps, other barriers may prevent people from identifying these symptoms as related to ePD. As a result, they may not seek timely consultations with healthcare providers (29, 30). Without a clearer understanding of which symptoms most significantly affect people living with ePD and the ways in which they do, there are limited opportunities to develop relevant clinical study endpoints to assess potential new interventions in clinical trial programs (31).

To better understand the impact of symptoms on the daily lives of ePD patients, this study aimed to identify the most common, burdensome, and worrisome symptoms from the patients’ perspective.

## Materials and methods

### Study design

This is a descriptive, cross-sectional study, with the primary objective of identifying the most common, burdensome, and worrisome symptoms experienced by patients living with ePD for 0–3 years (0 months to 48 months) and 4–5 years (48 months to 60 months) after diagnosis. This study also aimed to highlight any noteworthy differences in self-reported symptoms between these groups over the first 5-year after diagnosis. A secondary objective was to compare the reported frequency of common, burdensome, and worrisome symptoms reported by respondents in the United States (US) and Germany. These countries were selected to better understand any important differences in patient experiences across healthcare systems and to account for potential variations in access to care, support services, resources, and cultural influences. The respondent groups were selected in line with conventional clinical trial research practices in early-stage PD, which examines changes occurring within 5 years of diagnosis.

### Survey development

Based on previous studies (31), a survey was developed with three specific questions (see Supplementary Material) regarding (1) all symptoms that patients had experienced in the past or identified as part of their current disease experience (i.e., the most common symptoms); (2) the three most burdensome symptoms currently experienced, and (3) the three most worrisome symptoms when thinking about their future, regardless of whether they currently experience those symptoms. The survey was developed in collaboration with patient advocacy organizations in the US and Germany to ensure that the questions were framed in a patient-centered way, using clear and meaningful language. It was designed to maximize sensitivity to patients’ health status and minimize the burden on respondents (32, 33). The list of symptoms was based on six cardinal symptoms identified by Morel et al. (10). The study team selected eight additional symptoms through a targeted literature review (unpublished), resulting in a comprehensive list of 14 symptoms considered the most important and impactful from the perspective of ePD patients. These symptoms were included in the survey questionnaire (Supplementary Material) and were randomized for each respondent.

Data were also collected on age, gender, ethnicity in the US, and years since diagnosis. Ethnicity data were not collected in Germany because this information is not recorded there. Instead, the team used household income data in Germany to ensure a diverse and representative study sample.

### Sampling and recruitment

Using purposive and convenience sampling strategies (34), eligible survey respondents were sampled in the US and Germany. The inclusion criteria were as follows: (1) individuals aged 18–80 years, (2) residents of either the US or Germany, and (3) those who received a PD diagnosis within the past 5 years. A third-party recruiter (Fieldcats, Inc.) was engaged to recruit the survey participants and distribute the online survey. The recruiter used a combination of social media, online patient communities, medical support groups, healthcare facilities, and patient advocacy groups to reach out to the targeted patients in both countries. The recruiter ensured that participants met the eligibility criteria before sharing the survey link and sought medical documentation confirming a PD diagnosis within the last 5 years. Invitations to complete the survey were sent electronically through the above network channels. After answering three short pre-screening questions to determine eligibility, qualified participants completed the self-administered survey electronically using Decipher (by Forsta), a survey platform.

Survey responses were de-identified. The recruiter collected participants’ names and contact information for compensation purposes; however, the research team was not privy to this information. All procedures pertaining to participant privacy and confidentiality were strictly adhered to following IRB approval. Incentives were USD50 for American participants and EUR35 for German participants. Compensation details were not highlighted in the printed advertisement but were offered once interested participants contacted the recruiting partner. Compensation was provided at the end of the research.

### Data collection and analysis

Data was collected in August 2023. All survey results were self-reported based on patients’ subjective symptom experiences.

Using R software, descriptive statistics were used for the data analysis. Descriptive analyses included numerical and percentage frequencies for patients diagnosed for 0–3 years and 4–5 years, respectively. Stratified analyses by years since diagnosis and country of residence were conducted as well. Consistency of reporting by years’ post-diagnosis was analyzed by assessing symptoms frequency each year after diagnosis, without grouping patients into predefined categories of 0–3 years and 4–5 years.

As no formal hypothesis tests were specified prior to the start of the survey, no sample size or power calculations were done, and no hypothesis testing with statistical analysis was planned. Based on prior research by the study team, a sample size of 150 respondents (75 per country) was deemed a feasible target, given the challenges of accessing patients in the earliest stages of PD.

### Ethical review and approval

Between 1 May 2023 and 27 July 2023, the study received ethical approval for research to be conducted in both the US and Germany from Solutions IRB, a U.S.-based agency that oversees the ethical conduct of international research studies (protocol ID #2023/03/24 and 2023-Nov-0165). Respondents provided informed consent prior to completing the survey. The study was performed in accordance with the Declaration of Helsinki of 1964 and its later amendments. All researchers received ethics certification for working with human subjects in accordance with the Collaborative Institutional Training Initiative (CITI Program) or the Tri-Council Policy Statement (TCPS).

## Results

### Respondent characteristics

A total of 150 people living with ePD participated in the survey, with 75 patients from each participating country. A total of 106 (71%) survey participants were diagnosed for 0–3 years, and 44 (29%) were diagnosed for 4–5 years, with no differences between countries. The mean age of participants in the study was approximately 65 years and was similar between countries. Participants in the 0–3-year diagnosis group were approximately 5 years younger than participants in the 4–5-year diagnosis group. The majority of the respondents were male (85%), and, in the US, White/Caucasian (56%; Table 1).

**Table 1:** Demographic characteristics of survey participants.

	All (*n* = 150)	0–3 years since PD diagnosis	4–5 years since PD diagnosis
Participants (*n*)	n(%)	n(%)	n(%)
USA	75 (50%)	53 (71%)	22 (29%)
Germany	75 (50%)	53 (71%)	22 (29%)
Mean age, years (SD)	n(SD)	n(SD)	n(SD)
USA	64.97 (4.01)	63.42 (3.60)	68.73 (1.91)
Germany	64.77 (3.85)	63.19 (3.33)	68.59 (1.82)
Gender	%	%	%
Male	85%	81%	95%
Female	15%	19%	5%
Race/ethnicity, US-only	n(%)	n(%)	n(%)
White/Caucasian (non-Hispanic)	56 (75%)	36 (68%)	20 (91%)
Black/African American (non-Hispanic)	6 (8%)	5 (9%)	1 (4.5%)
Asian	10 (13%)	9 (17%)	1 (4.5%)
Hispanic/Latino	3 (4%)	3 (6%)	0 (0%)

### Commonness of symptom domains in all patients diagnosed with PD

**Figure 1:**
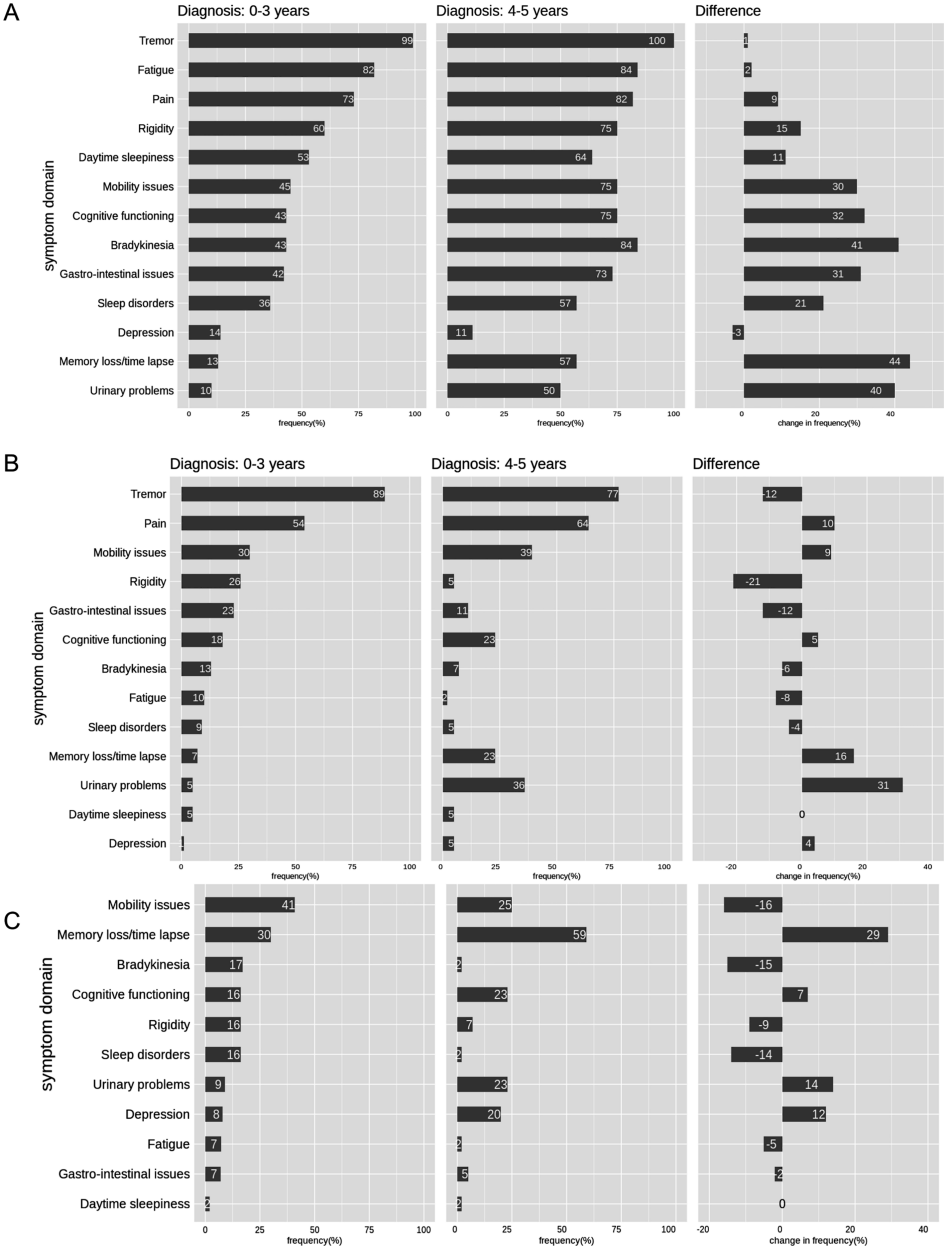
Frequency of symptom domains in patients diagnosed with PD for 0–3 years and 4–5 years **(A)** common, **(B)** burdensome, and **(C)** worrisome symptom domains.

Patients with ePD 0–3-year post-diagnosis reported considerably lower symptom frequency compared to those 4–5-year post-diagnosis, overall. Among patients 0–3-year post-diagnosis, tremor (99%), fatigue (82%), and pain (73%) were the three most frequently reported symptoms. Among patients 4–5-year post-diagnosis, tremor (100%), bradykinesia (84%), and fatigue (84%) were most frequently reported as common (Figure 1A).

Patients 4–5-year post-diagnosis reported pain (82%), rigidity (75%), mobility issues (75%), and cognitive impairment (75%) more frequently than patients 0–3-year post-diagnosis (73, 60, 45, and 43%, respectively).

The greatest symptom differences (i.e., increases greater than 30%) between patients 0–3 years and 4–5-year post-diagnosis were memory loss/time lapse (13% vs. 57%), bradykinesia (43% vs. 84%), and urinary problems (10% vs. 50%), followed by cognitive impairment (43% vs. 75%), gastrointestinal issues (42% vs. 73%), and mobility (45% vs. 75%).

Supplementary Tables 1–3 show 95% confidence intervals for the difference in percentages for each symptom between patients 4–5-year post-diagnosis and patients 0–3-year post diagnosis.

### Most burdensome and worrisome symptoms among all respondents

When comparing the two analyzed groups, the most frequently reported burdensome and worrisome symptom was tremor (89 and 77% in burdensome; 77 and 70% in worrisome; Figures 1B,C).

Pain was the second most reported burdensome (54%) and worrisome (54%) symptom among those 0–3-year post-diagnosis, and the third most reported worrisome symptom (57%) among those 4–5-year post-diagnosis (Figures 1B,C).

Among patients 4–5-year post-diagnosis, memory loss/time lapse was the second most frequently reported worrisome symptom (59%) and was much more frequently reported than among those 0–3-year post-diagnosis (30%; Figure 1C).

The greatest symptom difference (i.e., increase) in burdensomeness between 0–3- and 4–5-year post-diagnosis groups was urinary problems (5% vs. 36%), followed by memory loss/time lapse (7% vs. 23%), and pain (54% vs. 64%; Figure 1B).

The greatest symptom difference (i.e., increase) in worrisomeness between 0–3 and 4–5-years post-diagnosis groups was memory loss/time lapse (30% vs. 59%), followed by urinary problems (9% vs. 23%) and depression (8% vs. 20%; Figure 1C).

Rigidity was reported substantially less frequently as burdensome among patients 4–5-year post-diagnosis compared to patients 0–3-year post-diagnosis (5% vs. 26%), while mobility issues were reported to be much less frequently as worrisome at 4–5-year post-diagnosis (25% vs. 41%; Figures 1B,C).

### Frequency of symptoms by years since diagnosis

High consistency in symptom domain frequency reporting was observed over time (Supplementary Figures 1A–C), confirming that reported symptom burden gradually increases, in line with the analysis of the grouped categories.

### Differences between countries

High similarities were observed between participants from the US and Germany. Tremor was the most frequently reported symptom in both countries in the 0–3 years (100 and 98%) and 4–5 years (100 and 100%) post-diagnosis groups (Supplementary Figure 2A).

Differences between countries were also observed. Among German respondents 0–3-year and 4–5-year post-diagnosis, daytime sleepiness was the third most frequently reported symptom (83 and 86%), compared to ranking tenth (0–3 years, 23%) and twelfth (4–5 years, 41%) most frequently reported symptom in the US (Supplementary Figure 2A).

Among US respondents, rigidity was the third most frequently reported burdensome symptom at 0–3-year post-diagnosis (38%), compared to sixth (15%) most frequently reported burdensome symptom among the same group in Germany (Supplementary Figure 2B).

German respondents at 4–5-year post-diagnosis most frequently reported memory loss/time lapse as worrisome (77%), while US respondents in the same group reported this symptom third most frequently (41%; Supplementary Figure 2C).

## Discussion

Tremor was the most common and recognized motor symptom, which confirms other findings that tremor remains among the most relevant and most bothersome ePD symptoms in this patient population (10, 18). Tremor was reported by all survey participants, suggesting that our study population may not be representative of ePD patients who do not experience bothersome tremor symptoms. Clinically, ePD can be subtyped based on dominant motor features. In representative incident cohorts, the tremor-dominant (TD) subgroup accounts for 52–61% of cases, while the non-tremor-dominant/postural instability gait disorder (PIGD) subgroup comprises 27–36% (35, 36). TD patients typically have a younger onset, slower disease progression, milder non-motor symptom burden, and better cognitive outcomes. In comparison, PIGD/non-TD patients exhibit older onset, more rapid progression, higher prevalence of severe non-motor symptoms (notably, cognitive impairment, apathy, and autonomic dysfunction), and worse prognosis (37, 38). Levodopa and deep brain stimulation may be limited in controlling tremor in some people living with PD, leading to increased stress, anxiety, and diminished quality of life even early on in patients’ illness trajectory (18). Non-TD does not mean a complete absence of tremor, as it is very common among people with ePD, reported in approximately 90% of cases (39, 40). Tremor thus presents as an important patient-centered target for evaluation in interventional symptomatic clinical trials in a diverse and representative ePD population.

Increases in the reported frequency of common, burdensome, and worrisome symptoms over time provide key insights into what matters most to patients. Among ePD respondents, the top five symptoms reported to increase in frequency between the 0–3 and 4–5-year post-diagnosis are memory loss/time lapse, cognitive impairment, bradykinesia, mobility issues, and urinary and gastrointestinal problems. These findings build on the findings of Morel et al. (10), who demonstrated not only which symptoms are most meaningful to patients living with ePD because they are burdensome and worrisome, but additionally, which of these symptoms increased in reported frequency and thus should be focused on as increasing in meaningfulness to patients in the first 5 years. Among respondents 0–3 years post-diagnosis, memory loss and cognitive impairment were not reported as being common, yet they were reported as being worrisome. Conversely, fatigue was reportedly quite common among respondents, though it was not rated most often as burdensome or worrisome. These findings suggest that patients may view lesser common symptoms as priorities for intervention. In contrast, more common but less burdensome symptoms may be attributed less urgency.

In addition, participants frequently reported pain as one of the most burdensome symptom domains. It is important to note that pain is often multifactorial (as are other symptoms) and may or may not be immediately viewed as related to PD. Available clinical outcome assessment questionnaires used to measure pain in ePD in interventional clinical trials are often not fit for purpose, having been validated only in later stages of PD. This makes it difficult to discern the source of pain and its relationship to other PD symptoms, such as rigidity or depression (41, 42). The current study has shown that pain, along with other non-motor symptoms, is more generally important to patients, a finding that is well supported in the literature. However, non-motor symptoms are difficult to measure and are often neglected clinically, attributable in part to patients’ underreporting and in part to clinicians’ attention to more observable and measurable symptoms (43, 44). Further investigation of patients’ lived experience with non-motor symptoms in ePD may help identify tools to capture best and qualify the burden they present in everyday life.

Finally, notable differences between German and US respondents in the reported frequency of certain symptoms suggest that the ePD experience may differ between these two countries. Prior research has demonstrated ethnic variation in PD prevalence and symptoms as well as the influence of socioeconomic factors and health inequalities in PD patient diagnosis and illness experiences (20, 45). This study found that German respondents 4–5-year post-diagnosis more frequently reported non-motor symptoms as common (cognitive impairment) and worrisome (memory loss/time lapse) compared to US respondents. These differences may be explained by cultural differences in how individuals perceive the relative importance of their symptoms and the impact those symptoms have on individuals’ roles in society, for example, whether they are very physically active or continue to work full-time. This observation suggests that geographically specific symptom frequency may be important for further exploration, as it might impact outcome measurements in clinical trials and draw attention to how patients experience their disease differently within the context of a specific health system.

## Limitations

This survey has several limitations. The cross-sectional study design allowed for the collection of data from this patient population only at a specific point in time and did not allow for tracking any symptom changes in individuals over time, as it would have been done within a longitudinal study design such as that used in the Norwegian ParkWest study or the Parkinson’s Progression Markers Initiative (46, 47). Healthy individuals, or patients not suffering from ePD, were not included as participants in the survey, which limits the possibility of discriminating between general age-related deterioration in the elderly and PD-related worsening in the affected patients. The current study did not elicit the type of pain identified as most burdensome to patients, for example, whether the pain experienced was of musculoskeletal or neuropathic origin, nor did it ask survey participants about potential current treatments and how actual therapies might impact the reported frequency of symptoms. Furthermore, no information was gathered on respondents’ current knowledge of common PD symptoms experienced at various stages, therefore providing insights into what they might reasonably be worried about in the future.

The generalizability of the study results is limited by the sample size, which was respectable given the difficulties in recruiting participants with ePD. Still, it remains too modest to warrant broad-reaching conclusions. Furthermore, the population was recruited from only two countries and was largely White and male-dominant. Relying on self-reported questionnaires may introduce bias, as patients with certain symptoms may be more likely to respond. Additionally, the absence of data on non-tremor-dominant PD diagnosis may have contributed to potential reporting bias. No evaluation of sex-related differences in ePD was planned or carried out in this study, although this is an area of interest for future research. The study was not intended to include or exclude any subtype of the ePD population, nor to identify or analyze any subtypes. All symptom data were self-reported by patients and were not confirmed by a clinician. There was no grading of symptom severity, including tremor, which limits comparisons of the impact of different symptoms. The study team was aware of racial differences in PD diagnosis and deliberately sought out more people of color respondents, yet were not successful in recruiting a more race-balanced sample in the US (48, 49). However, a strength of our study is its alignment with the sample composition in prior ePD research. The mean age of participants in the 0–3-year-diagnosis group was approximately 63 years, which is consistent with expectations and experiences in other studies (50, 51). De Rijk et al. reported a mean age at diagnosis of approximately 60 years (52).

Finally, this research was designed as a descriptive, exploratory study; thus, no formal hypothesis tests were specified before the start of the survey. No sample size or power calculations were done. Survey results should be viewed as descriptive and hypothesis-generating.

## Conclusion

Among people living with ePD, tremor, mobility issues, and pain were the most frequently reported burdensome and worrisome symptoms. Bradykinesia, mobility issues, memory loss/time lapse, cognitive impairment, and urinary and gastrointestinal problems were reported with a frequency of at least 30% higher in the 4–5-year group. These survey results present robust and contemporary insights into the impact of ePD motor and non-motor symptoms on American and German survey participants’ daily lives when diagnosed with ePD. This study leverages knowledge of cardinal motor and non-motor symptoms of ePD to inform not only future clinical study designs but also increase awareness and knowledge of ePD symptoms that matter most to patients among healthcare professionals engaged in actual clinical practice.

## Data Availability

The datasets presented in this article are not readily available because the data is not publicly available as per Novartis’ data sharing policy. Requests to access the datasets should be directed to Simon Messner, simon.messner@novartis.com.
