# Supplementary material for: Most common, burdensome, and worrisome symptoms experienced by people living with early Parkinson’s disease in Germany and the United States—Results from a cross-sectional survey
Source: Front Neurol. 2026 Jul 8;17:1756255. doi: 10.3389/fneur.2026.1756255 (PMC13388077; doi:10.3389/fneur.2026.1756255)
Supplement: Supplementary file 1 [file Supplementary_file_1.DOCX]

## Supplementary Materials

**Survey Questions Presented to Study Participants**

| *Question* | **Which of the following symptoms have you experienced in the past or experience currently? Please select all that apply.** |
| --- | --- |
| *Answer type* | Multi-select |
| *Answer range* | 1. Bradykinesia/slowness (functional slowness) 2. Cognitive functioning (may include: attention/concentration, decision-making, disorganization, difﬁculties with multitasking, difﬁculties with reasoning/problem solving, and/or mental fog) 3. Day time sleepiness 4. Depression 5. Fatigue 6. Memory loss or time lapse 7. Mobility issues (particularly fine motor dexterity and/or subtle gait abnormalities) 8. Pain 9. Rigidity/stiffness 10. Tremor 11. Gastro-intestinal / constipation 12. Urinary problems 13. Sleep disorders 14. Sexual dysfunction / problems 15. Other (please specify) ____________________ |

###

| *Question* | **Of all the symptoms you listed in the last question, which three are the most burdensome right now? Please select no more than 3 symptoms.** |
| --- | --- |
| *Answer type* | Multi-select **(have only symptoms selected in Q7 appear)** |
| *Answer range* | 1. Bradykinesia/slowness (functional slowness) 2. Cognitive functioning (may include: attention/concentration, decision-making, disorganization, difﬁculties with multitasking, difﬁculties with reasoning/problem solving, and/or mental fog) 3. Day time sleepiness 4. Depression 5. Fatigue 6. Memory loss or time lapse 7. Mobility issues (particularly fine motor dexterity and/or subtle gait abnormalities) 8. Pain 9. Rigidity/stiffness 10. Tremor 11. Gastro-intestinal / constipation 12. Urinary problems 13. Sleep disorders 14. Sexual dysfunction / problems 15. Other ________ |

###

| *Question* | **When thinking about the future, what symptoms do you worry about the most? These could be symptoms you have right now or symptoms that you worry about getting in the future. Please select up to 3 symptoms that worry you the most.** |
| --- | --- |
| *Answer type* | Multi-select |
| *Answer range* | 1. Bradykinesia/slowness (functional slowness) 2. Cognitive functioning (may include: attention/concentration, decision-making, disorganization, difﬁculties with multitasking, difﬁculties with reasoning/problem solving, and/or mental fog) 3. Day time sleepiness 4. Depression 5. Fatigue 6. Memory loss or time lapse 7. Mobility issues (particularly fine motor dexterity and/or subtle gait abnormalities) 8. Pain 9. Rigidity/stiffness 10. Tremor 11. Gastro-intestinal / constipation 12. Urinary problems 13. Sleep disorders 14. Sexual dysfunction / problems 15. Other (please specify) ___ |

**Supplementary Tables:** Lower and upper confidence intervals for the difference between symptom frequency between patients diagnosed for 0-3 years versus 4-5 years with PD.

| Symptom | Percentage 0-3 years | Percentage 4-5 years | Difference | Lower 95% Confidence Interval for Difference | Upper 95% Confidence Interval for Difference |
| --- | --- | --- | --- | --- | --- |
| Bradykinesia | 43.40 | 84.09 | 40.69 | 26.35 | 55.04 * |
| Cognitive functioning | 43.40 | 75.00 | 31.60 | 15.71 | 47.50 * |
| Daytime sleepiness | 52.83 | 63.64 | 10.81 | -6.29 | 27.90 |
| Depression | 14.15 | 11.36 | -2.79 | -14.28 | 8.70 |
| Fatigue | 82.08 | 84.09 | 2.02 | -11.03 | 15.06 |
| Memory loss/time lapse | 13.21 | 56.82 | 43.61 | 27.62 | 59.60 * |
| Mobility issues | 45.28 | 75.00 | 29.72 | 13.80 | 45.64 * |
| Pain | 72.64 | 81.82 | 9.18 | -5.03 | 23.39 |
| Rigidity | 60.38 | 75.00 | 14.62 | -1.20 | 30.45 |
| Tremor | 99.06 | 100.00 | 0.94 | -0.90 | 2.78 |
| Gastro-intestinal issues | 41.51 | 72.73 | 31.22 | 15.06 | 47.38 * |
| Urinary problems | 10.38 | 50.00 | 30.62 | 23.75 | 55.50 * |
| Sleep disorders | 35.85 | 56.82 | 20.97 | 3.72 | 38.22 * |

**Table 1. Common symptoms**

| Symptom | Percentage 0-3 years | Percentage 4-5 years | Difference | Lower 95% Confidence Interval for Difference | Upper 95% Confidence Interval for Difference |
| --- | --- | --- | --- | --- | --- |
| Bradykinesia | 13.21 | 6.82 | -6.39 | -16.24 | 3.46 |
| Cognitive function | 17.92 | 22.73 | 4.80 | -9.57 | 19.18 |
| Daytime sleepiness | 4.72 | 4.55 | -0.17 | -7.53 | 7.19 |
| Depression | 0.94 | 4.55 | 3.60 | -2.82 | 10.03 |
| Fatigue | 10.38 | 2.27 | -8.10 | -15.39 | -0.82 |
| Memory loss/time lapse | 6.60 | 22.73 | 16.12 | 2.87 | 29.38 * |
| Mobility issues | 30.19 | 38.64 | 8.45 | -8.39 | 25.28 |
| Pain | 53.77 | 63.64 | 9.86 | -7.23 | 26.95 |
| Rigidity | 26.42 | 4.55 | -21.87 | -32.28 | -11.46 |
| Tremor | 88.68 | 77.27 | -11.41 | -25.18 | 2.37 |
| Gastro | 22.64 | 11.36 | -11.28 | -23.58 | 1.03 |
| Urinary problems | 4.72 | 36.36 | 31.65 | 16.87 | 46.42 * |
| Sleep disorders | 9.43 | 4.55 | -4.49 | -13.19 | 3.41 |

**Table 2. Bothersome symptoms**

| Symptom | Percentage 0-3 years | Percentage 4-5 years | Difference | Lower 95% Confidence Interval for Difference | Upper 95% Confidence Interval for Difference |
| --- | --- | --- | --- | --- | --- |
| Bradykinesia | 16.98 | 2.27 | -14.71 | -23.10 | -6.31 |
| Cognitive functioning | 16.04 | 22.73 | 6.69 | -7.53 | 20.91 |
| Daytime sleepiness | 1.89 | 2.27 | 0.39 | -4.72 | 5.49 |
| Depression | 7.55 | 20.45 | 12.91 | -0.03 | 25.84 |
| Fatigue | 6.60 | 2.27 | -4.33 | -10.79 | 2.13 |
| Memory loss/time lapse | 30.19 | 59.09 | 28.90 | 11.95 | 45.86 * |
| Mobility issues | 40.57 | 25.00 | -15.57 | -31.41 | 0.28 |
| Pain | 53.77 | 56.82 | 3.04 | -14.40 | 20.49 |
| Rigidity | 16.04 | 6.82 | -9.22 | -19.43 | 0.99 |
| Tremor | 77.36 | 70.45 | -6.90 | -22.56 | 8.76 |
| Gastro-intestinal issues | 6.60 | 4.55 | -2.06 | -9.82 | 5.70 |
| Urinary problems | 9.43 | 22.73 | 13.29 | -0.28 | 26.87 |
| Sleep disorders | 16.04 | 2.27 | -13.77 | -22.02 | -5.51 |

**Table 3. Worrisome symptoms**

**Note:** * indicates that the confidence interval for the difference excludes 0. For a particular symptom, this is equivalent to saying that the observed difference is significantly different from 0. However, there are 3x13 = 39 confidence intervals in Tables 1, 2 and 3 combined, and so claiming significance without adjusting for the fact that, in effect, 39 significance tests have been done, is not possible. It is quite likely that two (5%) of the “significant” confidence intervals in Tables 1, 2 and 3 combined do not imply a significant difference at the 5% level.

**Supplementary Figure 1.** Cross-sectional analysis of frequency of (A) common, (B) burdensome and (C) worrisome symptom domains (complete data set)

Tremor

Fatigue

Pain

Rigidity

Memory loss/ time lapse

Frequency (%)

of common symptoms

**A. Commonness**

**B. Burdensomeness**

Frequency (%) of three most worrisome symptoms

**C. Worrisomeness**

0 year

1 year

**Time since diagnosis (years)**

5 years

100

90

80

70

60

50

40

30

20

10

0

100

90

80

70

60

50

40

30

20

10

0

100

90

80

70

60

50

40

30

20

10

0

Mobility issues

Day time sleepiness

Sleep disorder

Cognitive functioning

Gastro intestinal

Bradykinesia

Depression

Urinary problems

Frequency (%) of three most burdensome symptoms

2 year

3 year

4 year

**Supplementary Figure 2.** Comparison between countries. Frequency of responses Cross-sectional analysis of frequency of (A) common, (B) burdensome and (C) worrisome symptom domains (complete data set)
